# Molecular and Physiological Diversity of Indigenous Yeasts Isolated from Spontaneously Fermented Wine Wort from Ilfov County, Romania

**DOI:** 10.3390/microorganisms11010037

**Published:** 2022-12-22

**Authors:** Viorica Maria Corbu, Ortansa Csutak

**Affiliations:** Department of Genetics, Faculty of Biology, University of Bucharest, 060101 Bucharest, Romania

**Keywords:** wine wort yeasts, biodiversity, MALDI-TOF, PCR-RFLP, sequencing, stress conditions, virulence and pathogenicity, antimicrobial, substrate competition, killer toxins

## Abstract

(1) Background: Wine yeast research offers the possibility of isolating new strains with distinct metabolic properties due to the geographical location of the vineyard and the processes used in winemaking. Our study deals with the isolation and identification of six yeasts from spontaneously fermented wine wort from Romania and their characterization as new potential starter culture for traditional beverages, for food industry or biomedicine. (2) Materials and methods: The isolates were identified using conventional taxonomy tests, phenotypic phylogeny analysis (Biolog YT), MALDI-TOF mass spectrometry, PCR-RFLP, and sequencing of the ITS1-5,8S-ITS2 rDNA region. The capacity of the yeasts to grow under thermal, ionic, and osmotic stress was determined. The safe status was confirmed by testing virulence and pathogenicity factors. Assays were performed in order to evaluate the growth inhibition of *Candida* strains and determine the antimicrobial mechanism of action. (3) Results and discussions: The yeast isolates were identified as belonging to the *Metschinikowia*, *Hanseniaspora*, *Torulaspora*, *Pichia*, and *Saccharomyces* genera. All the isolates were able to develop under the tested stress conditions and were confirmed as safe. With the exception of *S. cerevisiae* CMGB-MS1-1, all the isolates showed good antimicrobial activity based on competition for iron ions or production of killer toxins. (4) Conclusions: The results revealed the resistance of our yeasts to environmental conditions related to industrial and biomedical applications and their high potential as starter cultures and biocontrol agents, respectively.

## 1. Introduction

Wine production is a complex process that involves a wide variety of biotic and abiotic factors. An important parameter with a high impact on wine quality is the microbial community involved in sugar fermentation [1]. The grapes phyllosphere includes yeasts, bacteria, and filamentous fungal strains. Among the most common types of yeasts involved in the initiation of the alcoholic fermentation process are C*andida*, *Pichia*, *Kluyveromyces*, *Metschnikowia*, *Torulaspora*, *Saccharomyces,* and *Zygosaccharomyces*. Although alcoholic fermentation is mainly carried out by strains belonging to the *S. cerevisiae* species, other members of the microbial community intervene in the development of taste and aroma. For example, *Candida stellata*, *Kloeckera apiculate,* and *Meyerozyma guilliermondii* produce large amounts of glycerol to protect themselves from oxidative stress during alcoholic fermentation. This compound gives craft wine a light texture and complex taste. In addition, these types of yeast enrich the taste of wine by producing acetaldehydes, succinic acid, or different esters [2].

Even today, wine is obtained in households by spontaneous fermentation of sugars from grapes/cereals [3]. At an industrial level, starter cultures belonging to the *S. cerevisiae* species are used to control the fermentation process, but in the case of artisanal beverages, a particularly high emphasis is placed on preserving the authenticity and specificity of traditional beverages. In this case, the microbial community involved in spontaneous fermentation originates, in most cases, from the surface of the grapes [4].

The study of microorganisms involved in the spontaneous fermentation of food products offers the possibility of isolating new strains with high biotechnological potential, either for the optimization of biotechnological processes associated with the food industry (in the form of starter cultures) or for their use as a resource for obtaining compounds of industrial interest. The use of starters has become a frequently used technique in wine fermentation, even for artisanal beverage production. However, since artisanal beverage production involves respecting local specificity, it is desirable that these starter cultures be developed starting from strains from the local biodiversity. In order to select different microbial strains to be used as starter cultures, it is essential to have good fermentation kinetics, high tolerance to osmotic and ethanol stress, and a high ability to assimilate different nitrogen sources [5]. Although *S. cerevisiae* remains the main yeast species used for wine fermentation, at the beginning of the 21st century, non-*Saccharomyces* species have begun to take hold on the market [6]. As mentioned before, non-*Saccharomyces* species can be used in the winemaking process to develop specific aromas or traits. *Torulaspora delbrueckii* is the first non-*Saccharomyces* species used as a commercial wine starter culture. The species showed high potential for improving wine parameters such as low amounts of acetic acid and increased amounts of glycerol, mannoproteins, or polysaccharides. More than that, it is associated with increasing the amount of different aromatic compounds (lactones, thiols, or terpenes) simultaneously with the decrease of higher alcohols [6,7]. *Schizosaccharomyces pombe* is another yeast species commercialized nowadays as starter culture for wine production, being associated with deacidifying activity through malo-alcoholic fermentation [8,9]. Medina et al. [10] proved that sequential inoculation of *S. cerevisiae* starter culture and *H. vineae* strains produced relatively dry wines with increased aroma and flavour panel associated with higher concentrations of glycerol, acetyl, and ethyl esters.

However, when used in different industrial and biotechnological processes, the microorganisms, including yeasts, are exposed to stringent environmental conditions, such as extreme temperature variations, osmotic or ionic stress, and also strongly acidic or strongly alkaline pH values. 

The mesophilic microorganisms are most widely used in industrial processes, making it necessary to maintain a constant temperature in order to assure the best metabolic conditions. In addition, the environmental temperature change triggers modifications of the growth rate, delaying the entry into the exponential phase of growth, the production of enzymes involved in catabolic and anabolic processes, and, last but not least, can cause changes in the cell morphology [11]. For example, in fermentation, due to the chemical process itself, the temperature increases quickly. As a consequence, mesophilic microorganisms can face difficulties in their metabolic activity. Thus, it is necessary to decrease or increase the environmental temperature in order to maintain it in the range of values optimal for the growth of the microorganisms. From an economic point of view, lowering or increasing the temperature is expensive. A solution might be represented by the usage of thermophilic strains which might be isolated from spontaneously fermented food products [12]. 

The exposure of the microbial strains to osmotic stress is another characteristic of the processes in the food industry. Sodium chloride (NaCl) is the main salt used in food industry, for the development of specific flavors as well as a preservative. Since the presence of NaCl in the culture medium exerts both osmotic and ionic stress on yeast cells, they must tolerate it without losing their biotechnological potential.

Similarly, the stress induced by the variation of the pH value is a great challenge for the microbial resource during industrial processes. Yeasts are microorganisms that develop optimally at slightly acidic pH values (~6.2), but in order to withstand even lower values, they have acquired a series of defense mechanisms that involve changing the permeability of the cell membrane, the overexpression of genes involved in the synthesis of elements constituents of proton pumps, or the consumption of protons entering the intracellular environment in various metabolic processes. The decrease in the pH value is determined by the accumulation of organic acids in the culture medium, a situation often encountered in the food industry [13]. There is also a limited number of yeast strains able to grow at alkaline pH values, among which the strains from the genus *Candida* stand out [14]. 

The ability of yeasts to grow in oligotrophic conditions is a particularly important element from an industrial point of view, providing an advantage when they come into contact with mammalian organisms and potentially causing infections [15]. Therefore, besides their resistance to stress conditions, the safe status represents a major characteristic of the yeast strains successfully used in industry and biotechnology. Among the most important characteristics that can indicate the pathogenic potential of yeasts, the following stand out: the ability to secrete some hydrolytic enzymes such as proteases, DNases, and hemolysins; the ability to adhere to an inert substrate; and morpho-genetic dimorphism. 

An important feature of the spontaneously developed fermentative processes is that they take place without complete sterilization of the raw material. Therefore, it is considered that there is a continuous transfer of microorganisms between the food product and the ambient environment in which the microbial fermentation takes place [16,17]. This fact indirectly provides the possibility of analyzing the microbial biodiversity according to the geographical area. Preserving local specificity, including microbial specificity, allows access to new market sectors in the food industry focused on organic or traditional food products, thus increasing the economic value of the resource. In this context, the aim of the present study was the isolation and characterization of new yeasts from spontaneously fermented wine wort from Romania as a first step for their further use as potential starter cultures for traditional beverages and for different associated industrial and biotechnological fields.

## 2. Materials and Methods

### 2.1. Biological Material

For the isolation of yeasts, 10 mL of spontaneously fermented wine wort from Ilfov County, Romania, were taken and transferred into 50 mL sterile Falcon tubes. Subsequently, the sample (~1 mL) was brought to a final volume of 10 mL, and serial decimal dilutions were made in sterile physiological water up to 10^−8^. From the last 2 dilutions, 100 µL of suspension were spread on yeast peptone glucose agar (YPGA) medium (0.5% yeast extract, 1% peptone, 0.2% glucose, 2% agar) using a Drigalski spatula. The plates were incubated for 48 h at 28 °C. At the end of the incubation period, yeast colonies with a distinct morphological appearance were selected and purified by making successive passages on YPGA medium.

Six yeasts were isolated and maintained in a Revco LegaciTM Refrigeration System (Copeland, UK) at −70 °C on yeast peptone glucose (YPG) medium (YPGA medium without agar) supplemented with 20% glycerol. Before their use, yeasts were grown for 24 h at 28 °C on YPGA slants.

### 2.2. Conventional Taxonomy Tests

**The morphological aspect of the yeast colonies** was observed after 24–48 h growth on YPGA medium, using a stereomicroscope SZM-1 (Optika Microscopes, Ponteranica, Italy). **The cellular aspect and the budding type** were determined using an optical microscope (MICROS, Sankt Veit an der Glan, Austria). The presence of **sexual reproduction** was determined using two types of culture media. The first specific media contains CaCO_3_ at 10% to induce sporulation, and the second one is represented by 5% malt agar sporulation media [18]. After incubation for 5 days at 25 °C in darkness, the microscopic slides were visualized either directly using an optical microscope (40×) or stained with fuchsine 1/10 (for ascospores) and methylene blue 1% (for asci). The stained preparations were analyzed at an optical microscope (MICROS, Austria) using immersion oil [19]. As a positive control, the *S. cerevisiae* CMGB-RC diploid strain was used.

**Physiological and biochemical analyses** were performed according to Barnett et al. [20] and Kurtzman et al. [18]. **Aerobic assimilation of different carbon sources** (raffinose, D-galactose, and xylitol) was tested on yeast nitrogen base with aminoacids (YNB-Sigma Aldrich, St. Louis, MO, USA) (6.7 g/L) supplemented with 50 mM of each compound. From each culture, suspensions were made in sterile, demineralized water up to approximately 25 × 10^6^ cells/ mL. Each suspension was used to inoculate 1% into the testing tube containing different carbon sources. The growth was assessed using the card method [18]. As a positive control, we used test tubes with 50 mM glucose. For the negative control, no carbon source was added. 

**The ability to assimilate nitrogen sources** was tested using a similar protocol. The 10X yeast carbon base (YCB) (Sigma Aldrich) medium was supplemented with 1% stock solution: 7.8% KNO_3_, 2.6% NaNO_2_, 5.6% L-lysine, or 5.08% (NH_4_)_2_SO_4_ (positive control). The negative control did not contain a nitrogen source. However, for the interpretation of the results, it was taken into account the fact that a slight increase in yeast culture can also occur in the negative control due to atmospheric nitrogen diffusing into the culture media. 

**The ability to grow under an osmotic stress condition** was determined using YPGA medium supplemented with between 50 and 60% glucose. A yeast colony was seeded using the loop depletion technique on agar medium poured into Petri plates, followed by incubation at 28 °C. Plates were monitored daily for two weeks and the growth was quantified by reference to the positive control represented by the analyzed isolate cultivated on YPGA medium without glucose. 

**The ability to grow at different temperatures** was assessed by cultivating the yeasts on YPGA medium at 20, 28, 37, and 42 °C for two weeks. To avoid drying out the culture medium, each plate was sealed with parafilm tape. 

**Urease test** was performed using *Rhodotorula glutinis* CMGB-G1 as a positive control, *Yarrowia lipolytica* CMGB32 as a false positive control, and *S. cerevisiae* CMGB-RC as a negative control. For this test, Christensen’s urea agar supplemented with 0.1% of glucose was used. Positive results are indicated by the color changing from yellow to pink after 24–48 h of incubation [18,21]. Since false positives can occur, each positive isolate was also tested on Difco Urea Broth according to Kurtzman et al. [18]. The results were monitored every 30 min in the first 4 h of incubation at 37 °C and after 14 h. 

**Resistance to various cycloheximide concentrations** was determined on YNB medium supplemented with 50 mM glucose and 0.01% or 0.1% cycloheximide. For this purpose, 1.5 mL of fresh culture (cultivated for 24 h at 28 °C at 150 rpm in YPG broth) was centrifuged for 6 min. at 6500 rpm. The cellular sediment was used to prepare a cellular suspension of approximately 25 cells × 10^6^ cells/ mL in sterile distilled water. Each test tube containing culture media with cycloheximide was inoculated with 1% of the above-mentioned suspensions. The results were recorded every day for a week using the card method and *Candida boidinii* CMGB 95 as a positive control. 

### 2.3. Taxonomic Identification Using Automated Systems

For a more accurate taxonomic identification, the **Biolog Microbial ID System** was used following the manufacturer’s instructions. The inoculum and assays were processed according to the manufacturer’s instructions. Starting from fresh yeast cultures cultivated on YPGA medium for 24 h at 28 °C, a yeast inoculum was made in sterile water with a transmittance of 47, determined using the Biolog turbidimeter. A volume of 100 µL suspension was transferred to each well of a YT Biolog plate, followed by incubation at 28 °C for 3 days. The results were read at 24 h intervals using the MicroStation™ (Biolog, Inc., Hayward, CA, USA), and their interpretation was done using Microlog Software version 4.2 (Biolog, Inc., Hayward, CA, USA). The validation of the results and the working method was carried out by including in the study the reference strain *C. albicans* ATCC10231. 

For the **MALDI-TOF MS analysis**, the yeast isolates were grown overnight on YPGA medium at 28 °C and prepared for analysis according to the instructions provided by the manufacturer. The determination of the protein profile was performed using the Microflex III system (Bruker Daltonik, Bremen, Germany), and the results obtained were compared with existing profiles in the MALDI BioTyper Software database (Bruker Daltonics). The results were expressed in the form of scores in the range 0–3. Among the values obtained, only scores above the value 2 were considered relevant for identification at the species level [22,23].

### 2.4. Molecular Taxonomy Tests

#### 2.4.1. Genomic DNA Isolation

Genomic DNA was isolated using the protocol described by Csutak et al. [24]. The DNA samples were observed by agarose gel electrophoresis using 0.8% agarose, 0.5X Tris-Boric acid-EDTA (TBE). The concentration of the DNA samples was determined at OD = 260 nm with a NanoVue Plus spectrophotometer.

#### 2.4.2. PCR-RFLP and Sequencing of the ITS1-5.8S rDNA-ITS2 Region

The analysis of the ITS1-5.8S rDNA-ITS2 region was performed according to Csutak et al., [24]. The first step involved PCR amplification using specific primers ITS1 (5′-TCCGTAGGTGAACCTGCGG) and ITS4 (5′-TCCTCCGCTTATTGATATGC) (200 pM, TIB MOLBIOL). The amplicons were digested using four restriction endonucleases: *Cfo*I (5′-GCG/C-3′), *Hinf*I (5′-G/ANTC-3′), *Hae*III (5′-GG/CC-3′), and *Msp*I (5′-C/CGG-3′) (10 U/µL, Promega, Madison, WI, USA). The restriction fragments were observed by gel electrophoresis using 1.7% agarose and TBE 0.5X. The sizes of the amplicons and restriction fragments were determined using PyElph software [25]. Since for the isolates M1 and M5, the Biolog Microbial ID System and the MALDI-TOF MS analysis did not provide results, in order to confirm the data obtained using the conventional identification tests and the PCR-RFLP technique, the ITS1-5.8S rDNA-ITS2 amplicons were also sequenced. Sanger sequencing was performed by the CeMIA-Cellular and Molecular Immunological Applications Company (Larissa, Greece). The construction of contigs was done using the DNA Dragon 1.4.1 program [26]. Fasta sequences were uploaded on the GenBank database (Accession numbers: OP745444; OP745445). For the taxonomic identification of the isolates, the sequences obtained were compared with GenBank, using the BLAST algorithm [27]. 

### 2.5. Cell Growth under Stress Conditions

The yeast biomass accumulated under conditions of ionic, osmotic, and thermal stress and was spectrophotometrically quantified according to Corbu et al. [28].

To evaluate the impact of the pH value on microbial growth, the YPG medium with different pH values in the range of 3–12 was used. The pH adjustment was made with 2N HCl for pH < 6 values and with 40% NaOH for pH > 7 values. From cultures of 24 h at 28 °C on YPG medium, 1.5 mL were taken and centrifuged for 7 min at 7000 rpm. The cell pellet was washed twice with ADS, and then cell suspensions were made until the OD 600 nm was equal to 1. These suspensions were used to inoculate YPG medium with different pH values at a final concentration of 1% and a final volume of 300 µL. Cell growth was monitored spectrophotometrically 24 h after inoculation at 28 °C using a Synergy™ HTX Multi-Mode Microplate Reader. The positive control was represented by yeasts grown on YPG medium with an unadjusted pH (6.2).

To determine the growth capacity of medium with different NaCl conditions, cell suspensions were made according to the previously described protocol, but they were innoculated on YPG medium supplemented with NaCl in different concentrations from 0.5–12%. The positive control was represented by the yeasts grown on YPG medium without the addition of NaCl, and the growth was monitored spectrophotometrically after 24 h of incubation at 28 °C. 

In order to determine the capacity to grow at different temperature values, yeasts were cultivated on standard YPG medium according to the previously described protocol with the exception that in this case the incubation temperature was varied. Thus, after the inoculation of 1% yeast isolates in YPG medium, they were incubated for 24 h at 15 °C, 22 °C, 28 °C, 42 °C, and 60 °C. Taking into account that yeasts are generally mesophilic, the positive control was represented by the culture developed at 28 °C, and growth monitoring was also done spectrophotometrically by measuring the OD at 600 nm.

### 2.6. Confirmation of Safe Status—Determination of Virulence and Pathogenicity Factors

#### 2.6.1. Production of Hydrolytic Enzymes

The yeasts were cultivated on YPG medium for 24 h, 28 °C, 150 rpm. At the end of the incubation period, the cultures were centrifuged for 6 min at 6000 rpm, and the cell pellet was washed twice with sterile distilled water. The cell pellet resulting from the last centrifugation was resuspended in sterile water and diluted to an OD at 600 nm of 1. A volume of 10 µL of suspension was used as inoculum and spotted on specific media [29,30]. 

**Production of hemolysins** was determined using Sabouraud medium (Oxoid) (Hampshire, United Kingdom) supplemented with 5% defibrinated ram blood. The culture medium was prepared according to the manufacturer’s instructions, and the blood was added after sterilizing the medium (15 min at 121 °C). The positive result is indicated by the appearance of a clear area around the culture spots after 24–48 h of cultivation at 37 °C, which indicates the destruction of red blood cells from ram blood under the action of hemolysins. 

**Production of amylolytic enzymes** (amylases) was tested using YP medium (0.5% yeast extract, 1% bacteriological peptone, 1% NaCl) supplemented with 10% starch. After incubation for 48 h at 37 °C, the plates were stained by flooding with Lugol’s solution (50 g/L iodine and 100 g/L potassium iodide) for 3 min. Lugol solution causes the color of the culture medium in which the starch is found to turn from cream to deep blue. The positive result for amylase production is indicated by the appearance of clear areas around the culture spots.

**Caseinase synthesis** was tested using a similar method, with the YP medium supplemented with 10% casein from milk. The positive result is indicated by the formation of halos of white precipitate around the colonies. 

The presence of **siderophore-like compounds** was detected on plain agar medium (10 g/L bacteriological peptone, 1 g/L ammonium ferric citrate, 20 g/L agar, 18 g/L agar, pH 7.4) supplemented with 0.1% esculin. The ability to produce siderophores was observed as an intensely colored zone (brown to black) forming around the yeast colonies after 24 h of incubation at 37 °C.

**The production of DNases (extracellular deoxyribonucleases)** was analyzed using DNase Test Agar medium (Difco, East Rutherford, NJ, USA) supplemented after sterilization with 0.01% toluidine blue solution as a culture medium. The positive result was indicated by the appearance of light halos or pale pink areas around the yeast colonies. 

The ability to produce **phospholipases** was assessed using Sabouraud Dextrose Agar (Oxoid) supplemented with 10% egg yolk, 1.17 g/L NaCl, and 0.001% CaCl_2_. Egg yolk was added after sterilizing the medium according to the manufacturer’s instructions. The seeded plates were incubated for 72 h at 37 °C, and the positive result is indicated by the appearance of a white precipitate around the culture spots. 

Testing the production of **extracellular gelatinase-type proteolytic enzymes** involved the cultivation of yeasts on a specific medium with gelatin (1 g/L yeast extract, 5 g/L sodium taurocholate, 10 g/L bacteriological peptone, 0.1% NaCl, 30 g/L gelatin, and 15 g/L agar-agar). The plates were incubated for 48–72 h at 37 °C, and the positive result is indicated by the formation of a clear halo around the culture spots.

#### 2.6.2. Dimorphism Switching Assays

The morpho-genetic dimorphism was observed on RPMI 1640 medium (Roswell Park Memorial Institute) with L-glutamine, without sodium bicarbonate, supplemented with 10% (*v*/*v*) fetal bovine serum. The yeasts were cultivated on YPG medium for 24 h at 37 °C. The cultures were centrifuged for 5 min at 7000 rpm, and the resulting sediment was resuspended in RPMI 1640 medium in order to obtain suspensions with approximately 5 × 10^6^ cells/mL. After incubation for 3 h at 37 °C, the hyphal percentage was determined by counting the cells in hyphal/pseudohyphal form in 10 random microscopic fields and relating it to the total number of cells counted. The optical microscope with a 40X objective (MICROS, Austria) was used for the microscopic analysis [30].

#### 2.6.3. Evaluation of the Adhesion Capacity to Inert Substrate

The ability of each isolate to adhere to an inert substrate was determined following a protocol described by Jin et al. [31] and adapted by Sârbu et al., [30]. Thus, fresh yeast cultures grown on YPG medium for 18 h at 37°C and 150 rpm were centrifuged (7 min at 7500 rpm), and the cell sediment was washed twice with PBS (phosphate buffer saline buffer pH 7.4: 8 g/L NaCl; 0.2 g/L KCl; 1.42 g/L Na_2_HPO_4_; and 0.24 g/L KH_2_PO4). The cell pellet was resuspended in YNB medium with amino acids supplemented and 50 mM glucose to reach a cell density of approximately 1 × 10^5^ cells/mL. A volume of 200 µL from each yeast suspension was placed in each well of a 96-well plate. The plates were incubated for 72 h at 37 °C without shaking. At the end of the incubation period, the culture was discarded, and the wells were gently washed four times with PBS to remove non-adherent cells. The cells attached to the inert substrate were fixed with 100% methanol for 5 min, and after its removal, the biofilm was stained with 200 µL 1% crystal violet solution for 15 min. The excess dye was removed by washing four times with tap water. Subsequently, 220 µL of 33% acetic acid was pipetted into each well, and absorbance was determined at 490 nm using the Synergy™ HTX Multi-Mode Microplate Reader. The experiment was performed in triplicate and repeated three times. 

### 2.7. Qualitative Screening of Antimicrobial Activity 

#### 2.7.1. Substrate Competition Assays

The yeast isolates were tested for potential antimicrobial activity based on substrate competition against *Candida* pathogenic strains (*C. krusei* CMGB-Y8 and *C. parapsilosis* CMGB-Y3) isolated from vulvo-vaginal infections and potentially pathogenic strains (*C. albicans* ATCC10231, *C. parapsilosis* CBS604, and *C. krusei* CMGB94). For this purpose, the tested isolates were inoculated on YPGA medium and cultivated for 24 h. The *Candida* strains were grown overnight on YPG liquid medium at 28 °C and 150 rpm, then centrifuged for 7 min at 7000 rpm, and the cellular sediment was resuspended in sterile distilled water up to an OD 600 nm. A volume of 1. 2 mL of this suspension was evenly distributed on the surface of Petri dishes with YMA medium (3 g/L yeast extract; 10 g/L D-glucose; 5 g/L peptone; 3 g/L malt extract; 15 g/L agar), and excess liquid was removed. After drying the plates, a colony from the tested YPGA yeast cultures was spotted on their surface. The plates were incubated for 7 days at 28 °C and analyzed periodically. A positive result is indicated by the appearance of a halo around the spot of the tested yeast colonies, which is associated with growth inhibition of the *Candida* potentially susceptible strain inoculated on the surface of the Petri dish [32]. 

#### 2.7.2. Activity at Low pH Values

In this case, the suspensions from the potentially sensitive *Candida* strains (*C. parapsilosis* CMGB-Y3; *C. parapsilosis* CBS604; *C. albicans* ATCC10231; *C. krusei* CMGB94; and *C. krusei* CMGB-Y8) were obtained as mentioned above, and inoculated on the surface of Petri plates with K medium (phosphate citrate buffer 0.1 M pH 4.8, 20 g/L D-glucose, 10 g/L yeast extract, 10 g/L peptone, 20 g/L agar, 0.03–0.07% methylene blue). The yeast isolates investigated for antimicrobial activity at low pH values were grown on YPGA medium for 24 h at 28 °C, and the grown cultures were resuspended in ADS to an optical density equivalent of 3 McFarland. They were inoculated on the K medium as a spot (10 µL). Plates were then incubated at 28 °C and analyzed periodically for up to 7 days. The positive result is indicated by the appearance of a bright halo around the test isolates, which is associated either with the total inhibition of the growth of the potentially susceptible strain in the substrate or with a decrease in its multiplication rate [33].

### 2.8. Statistical Analysis

All experiments were done in triplicate, and the results obtained were expressed as mean ± SD (*n* = 3) using the Excel tool from the Microsoft Office 2016 package.

## 3. Results and Discussions

### 3.1. Taxonomic Identification

Food or drink products obtained through fermentation represent an important economic and cultural asset in most European countries. The isolation and characterization of microorganisms from these products and their use as a component part of the industrial winemaking process can represent a real calling card for the Romanian space. This approach allows for the preservation of local specificity and offers the possibility of developing new market segments by approaching the production of organic and/or traditional drinks or fermented food products [34]. In this context, the isolation of new yeast strains from food products obtained by spontaneous fermentation is one of the first steps necessary for building a collection of microorganisms that immediately responds to the needs of the Romanian community. 

**Figure 1:**
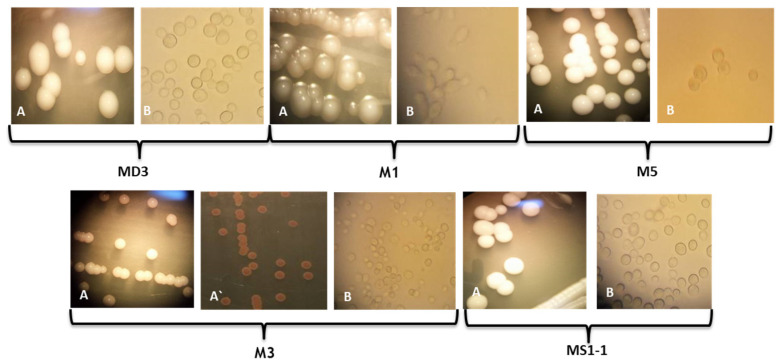
Appearance of colonies (A-averse; A` reverse) and cells (40×) (B) formed by yeasts isolated from wine wort (at 48 h; 28 °C; YPGA medium).

In our work, samples were taken from spontaneously fermented wine wort from Ilfov County, traditionally processed without the addition of preservatives or microbial starter cultures. The samples were directly diluted and inoculated on Petri dishes with an agar medium. Six colonies with distinct morphological appearances were selected and purified by successive passages on YPGA medium, as described in the Materials and Methods section. The isolate CMGB-M6 has been identified in a previous study as belonging to *P. kudriavzevii* [21]. The rest of the isolates were identified using both conventional and molecular techniques. Figure 1 presents the morphological aspects of each colony selected for purification. The appearance of the yeast colonies varies from white to cream, with the exception of the M3 isolate, for which red-brown pigmentation was observed near the colonies (Figure 1, A`). Such a situation is frequently encountered among strains from species such as *M. pulcherrima* and *P. membraniefaciens*, which produce iron-based pigments involved in substrate competition [35,36]. 

The colonies also vary in terms of the texture, shape, and appearance of the edge. Colonies with an entire edge (Figure 1, MD3; MS1-1, M3, M5), but also a flattened edge with a convex center (Figure 1, M1). Regarding the texture of the colonies, all of them have a smooth appearance, even after several days of incubation. The cells are round or oval in shape and show unipolar budding (Figure 1, B).

Conventional taxonomy tests are particularly relevant both in the classification at the genus and species level, as well as the description of metabolic and physiological characteristics relevant for potential biotechnological applications. Therefore, we selected the tests with the lowest level of difficulty, the highest power of interspecific discrimination, and the ability to provide additional information with metabolic value. The selected tests aimed to determine physiological characteristics such as the capacity for aerobic assimilation of carbon or nitrogen sources, the capacity for growth under conditions of osmotic stress induced by high concentrations of glucose, growth at non-permissive temperatures, urea degradation, and cycloheximide resistance. The results are presented in Table 1.

**Figure 2:**
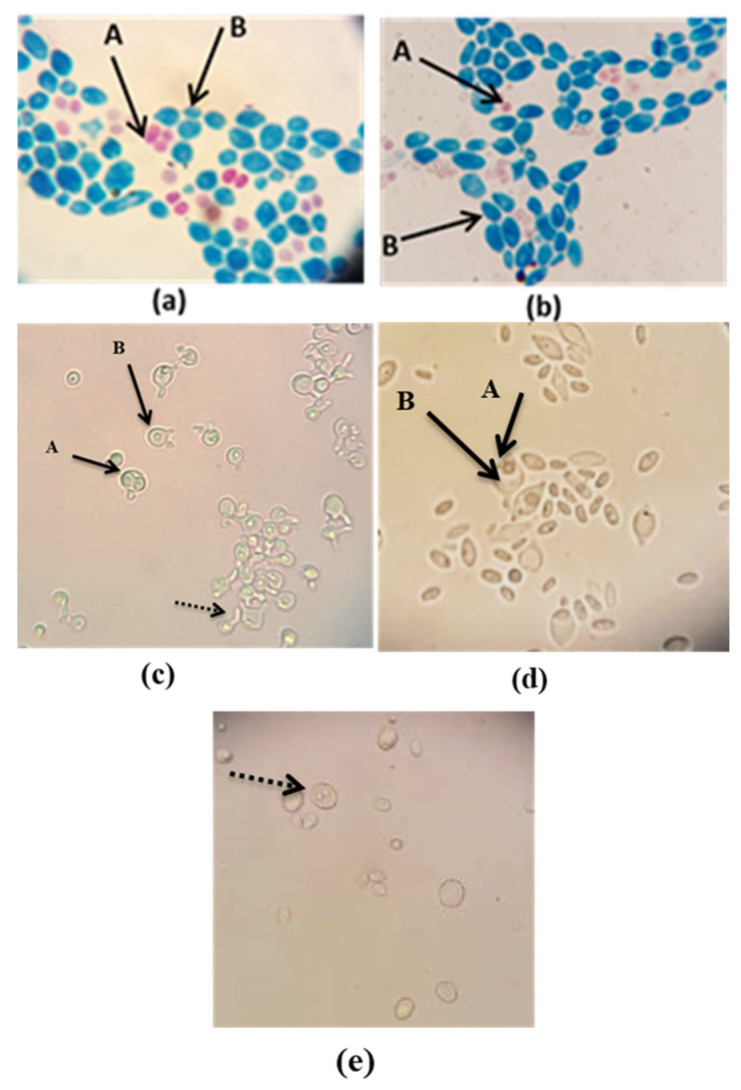
Highlighting the ability to form ascospores on medium with calcium carbonate as a non-fermentable carbon source (**a**,**b**) (100×), using specific staining with fuchsin and methylene blue or 5% malt agar medium (**c**–**e**); (**a**)—MD3; (**b**)—MS1-1; (**c**) M5; (**d**) M1; (**e**) M3. A: ascospore; B: ascus; (40×); dashed arrow indicates specific cellular modifications.

The tests regarding sexual reproduction (Figure 2) revealed that the MD3 and MS1-1 isolates produce two or three round-shaped ascospores/ascus. No ascospores were observed for the other three isolates when cultivated on CaCO_3_ media. The evaluation of sexual reproduction capacity by spore formation is a particularly important taxonomic criterion. Although not exhaustive, the observation of spores formed as a result of cultivation under conditions that prevent asexual reproduction of diploid cells is a criterion for taxonomic validation. Calcium carbonate medium is frequently used to induce ascospore formation in *S. cerevisiae* strains. Therefore, this test is not one of exclusion but of selecting the isolates that are most likely part of the *S. cerevisiae* species with the greatest degree of spread in the world of yeasts. Since M1, M5, and M3 isolates did not produce ascospores when cultivated on carbonate media, an additional sporulation medium was also used. On 5% malt agar medium, M1 and M5 isolates produced ascospores after 5 days of incubation at 25 °C. In the case of M5 isolates, ascospores have a round shape, and the ascus, as described in [18], presents a protuberance similar to a conjugation tube. (Figure 2c). In the case of M1, as it can be observed in Figure 2d, ascospores have a round shape, and each ascus contains one or two ascospores. The aspect of the ascospores formed is in accordance with the description in the specialized literature [18]. For the M3 isolate, no ascospores were identified on Malt agar medium, but specific cell structures were observed (Figure 2e). According to Kurtzman et al. [18], this type of cell can be chlamydospores or pulcherrima cells with lipid droplets, which are specific to members of the *Metschinikowia* species. Further studies will involve searching for new types of culture media, such as V8 juice agar, to induce sporulation in the case of the M3 isolate.

No detectable increase was recorded, with the exception of the isolate M3 which assimilated D-glucose, D-galactose and xylitol. None of the isolates assimilated potassium nitrate and sodium nitrite as nitrogen sources, but M1, M3, and M5 assimilated L-lysine. 

Urea degradation represents a particularly important taxonomic criterion, used to facilitate the discrimination of ascomycetes and basidiomycetes. When tested on agarized urea culture media, only the M3 isolate recorded a positive result during the first 48 h of incubation (Figure S2D). However, since in the case of Christensen’s urea agar, false positive results can also be registered (Figure S2A) [37,38], we decided to test the isolates on the urea broth medium as well. In this situation, the monitoring was carried out 14 h after the inoculation, and the M3 isolate recorded a negative result (Figure S3C).

**Figure 3:**
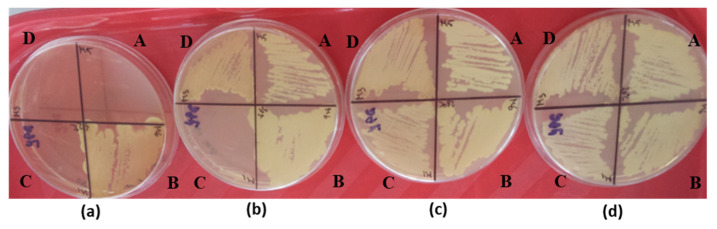
Qualitative assessment of the ability to tolerate thermal stress in A-M5, B-MS1-1, C-M1, and D-M3 after 7 days of incubation at 42 °C (**a**); 37 °C (**b**); 28 °C (**c**); and 20 °C (**d**).

The tests for resistance to stress conditions showed that all our isolates grew at temperatures in the range of 20–37 °C, with M1 showing significantly weaker growth at higher temperatures compared to its co-fermenters (Figure 3). In addition, none of the isolates was affected by osmotic stress induced by high concentrations of glucose.

The determination of resistance to different concentrations of cycloheximide is also a taxonomic criterion used for the distribution of yeast species into three distinct categories according to the minimum inhibitory concentration. All the yeast isolates tested were included in the cycloheximide sensitive strains category, inhibited by 1 µg/mL cycloheximide.

Using the Biolog System and Maldi-TOF mass spectrometry, a preliminary taxonomic classification of the newly isolated yeasts could be achieved. Both automated systems generated a result for three of the five isolates tested, except for isolates M1 and M5 when using the Biolog Microbial ID System, and isolate M5 when using Maldi-TOF (Table 1). It is interesting that the isolate MD3 was placed among the isolates belonging to *S. boulardii* using the Biolog Microbial ID system, while according to Maldi TOF it was determined as a *S. cerevisiae* strain (Figure S1). From a taxonomic point of view, there is a divided conception, with some researchers considering *S. boulardii* to be a variety of the *S. cerevisiae* species with probiotic properties, while others consider them to be a completely different species of the *Saccharomyces* genus. Between the two conceptions lie a series of conventional tests that can differentiate the two groups of yeasts. More precisely, according to recent studies, *S. boulardii* does not have the ability to form ascospores under nutrient-restrictive conditions and exhibits a greater intrinsic resistance to variations in culture parameters, such as low pH values, thus ensuring its survival and vegetative multiplication [39,40,41]. In the present situation, since the MD3 isolate was able to form ascospores on medium with calcium carbonate, it was preliminarily considered to be *S. cerevisiae*. As for the other four isolates, the profiles obtained using conventional tests were compared with those available in the literature (Table 2). Thus, the profile described for the isolate M3 is similar to that described for the reference strain of the *M. pulcherrima* species; that for the isolate MS1-1 is similar to *S. cerevisiae*; and that for the isolate M1 could be identified as *H. uvarum*. Although the M5 isolate could not be associated with any species using automated systems, its biochemical profile was compared to that of different yeast species that are frequently encountered in wine fermentation. Among them, the *T. delbrueckii* species proved to be the most suitable since it is characterized by a special metabolic versatility and is strongly influenced by the isolation source of the tested isolates [20,42]. 

**Figure 4:**
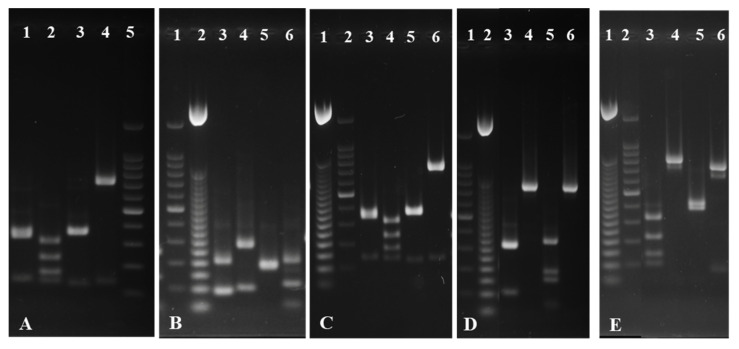
RFLP PCR profile of the ITS1-5.8SADNr-ITS2 region obtained for yeasts isolated from spontaneously fermented wine wort ((**A**): MS1-1; (**B**)-M3; (**C**)-MD3; (**D**)-M1; (**E**)-M5). For each isolate, the restrictions were loaded in the following order: *Cfo* I; *Hae* III; *Hinf* I; *Msp* I; (**A**)-5; (**B**)-1; (**C**)-2; (**D**)-1; (**E**)-2—100-bp DNA Ladder (ThermoFisher Scientific); (**B**)-2; (**C**)-1; (**D**)-2; (**E**)-1—BenchTop-50-bp-DNA Ladder (Promega).

In order to confirm the preliminary results obtained, we performed PCR-RFLP analysis of the ITS1-5,8SADNr-ITS2 region (Figure 4). The restriction reactions were carried out using four endonucleases: *Cfo*I, *Hae*III, *Hinf*I, and *Msp*I, and the profiles obtained were compared with data from the specialized literature (Table 3). The size of the resulting amplicons varied between 390 bp in the case of isolate M3 and 850 bp in the case of isolates MS1.1 and MD3. The endonucleases *Cfo*I and *Hinf*I generated the largest number of fragments and could be considered most suitable for possible further biodiversity studies on strains originating from fermented products. On the contrary, no restriction fragments could be observed for the isolates M1 and M5 upon using *Hae*III and *Msp*I in the restriction reaction (Figure 4—D4 and D6 and E4 and E6, respectively). 

The comparative analysis of the restriction profiles obtained from the isolates with those from similar studies confirmed that the isolates MD3 and MS1-1 belong to the *S. cerevisiae* species, M1 to *H. uvarum*, and M3 to *M. pulcherrima*. 

The ITS-RFLP technique was successfully used by other researchers as a molecular technique to identify yeasts involved in different fermentation processes, such as brewing yeast species [49]. Moreover, Silva-Barbedo et al. [51] recommend the PCR-RFLP technique applied to the ITS1-5.8S-ITS2 region as a reliable method to be used in clinical mycology laboratories to identify yeast strains. Although this technique has some disadvantages, since the results obtained by conventional tests (Biolog and MALDI TOF )were confirmed by RFLP-PCR for the isolates M3, MD3, and MS1-1, their taxonomic classification can be considered accurate.

Since for the isolates M1 and M5, no identification was obtained using the Biolog system and/or MALDI-TOF MS, we decided to sequence the ITS1-5,8SADNr-ITS2 amplicons. The sequences obtained for the isolates M1 and M5 were compared with those in the NCBI database and introduced with the accession numbers OP745444 for M1 and OP745445 for M5. The sequencing analysis confirmed M5 as belonging to the *T. delbrueckii* species, a species frequently associated with the development of a specific fruity aroma during wine fermentation processes [5,7,52].

### 3.2. Characterization of Industrial and Biotechnological Potential

#### 3.2.1. Growth under Stress Conditions

The next step in the present study was to determine growth and biomass accumulation under various stress conditions, and to confirm the safe status of all six yeasts isolated from wort, including the previously identified *P. kudriavzevii* CMGB-M6.

##### Influence of Thermal Stress

**Figure 5:**
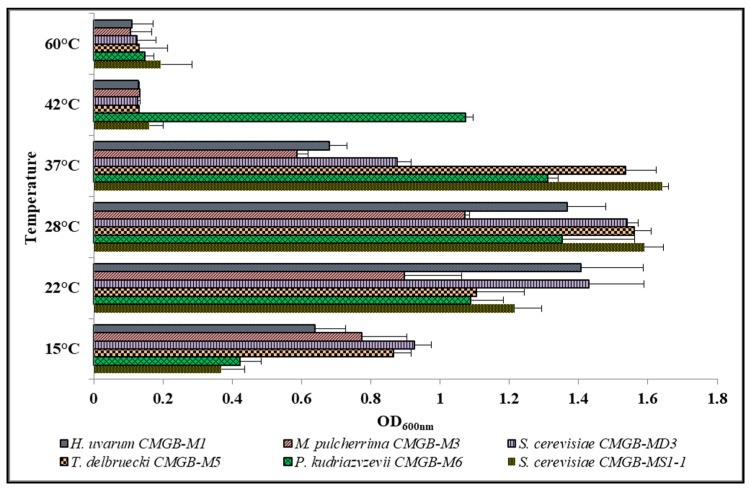
Growth rate of yeasts isolated from wort sediment after 24 h of cultivation at different temperature values.

The ability of our six new yeasts to grow at various temperatures was monitored over a 24-h interval by measuring the optical density at 600 nm. All the isolates grew at temperatures between 22–37 °C, with the optimal temperature value being 28 °C (Figure 5), with the exception of *P. kudriavzevii* CMGB-M6, for which significant growth was also recorded at 42 °C. The members of the *P. kudriavzevii* species present as the main mechanism of resistance to thermal stress, the accumulation of trehalose and glycerol. In fact, their ability to tolerate temperatures higher than 37 °C recommends them for a series of industrial processes aimed at obtaining valuable compounds for the food and cosmetic industries, as well as for biomedical applications [53,54].

The behavior of the two *S. cerevisiae* isolates (MD3 and MS1-1) is slightly different. Although both recorded best growth at 28 °C, *S. cerevisiae* MD3 preferred lower growing temperatures while *S. cerevisiae* MS1-1 grew at higher temperatures. This suggests that *S. cerevisiae* CMGB-MD3 could be further used in the first stages of wine fermentation, being able to develop at fairly low temperatures, while *S. cerevisiae* CMGB-MS1-1 could be used in the late stages, when the temperature in the fermentation tank increases. For *M. pulcherrima* CMGB-M3, the optimal growth temperature is between 15 and 37 °C. Pawlikowska et al. [55] proved similar properties for a *M. pulcherrima* strain isolated from flowers and fruits. More than that, the above-mentioned study showed that different members of this species can grow even at 2 °C, which, in correlation with the antifungal potential of *M. pulcherrima* strains, is highly valuable for preventing fungal contamination of fruits stored at low temperatures. *H. uvarum* CMGB-M1 isolate was able to grow at temperature between 15 and 37°C which is confirmed in a study performed by Salvado et al. [56] in which other yeasts isolated from wine fermented products are characterized. 

##### Influence of Osmotic Stress

**Figure 6:**
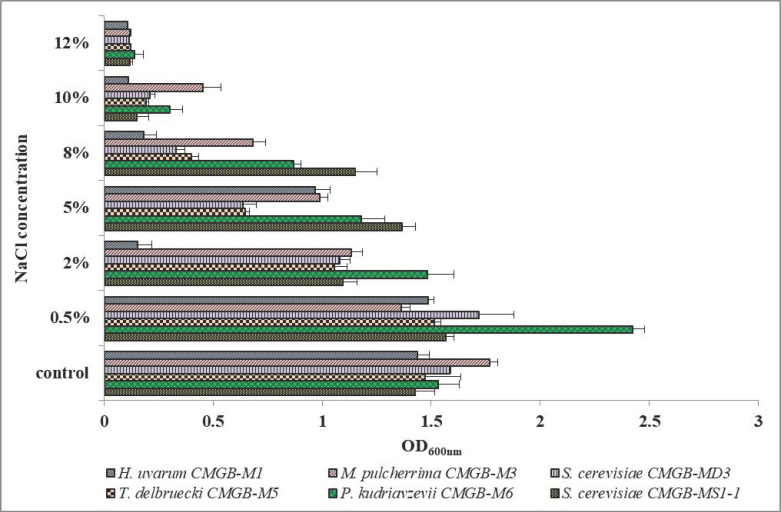
Growth rate of yeasts isolated from wort sediment after 24 h of cultivation in the presence of osmotic stress induced by NaCl at different concentrations.

The ability of the yeasts to grow in the presence of different concentrations of NaCl was determined by monitoring the DO 600 nm variation 24 h after inoculation. According to Figure 6 most of the isolates tolerated small concentrations of NaCl. Moreover, all the isolates recorded a higher growth rate in the presence of 0.5% NaCl compared to the control (YPG medium without NaCl), except for the isolate *M. pulcherrima* CMGB-M3. However, *M. pulcherrima* CMGB-M3 tolerated up to 10% NaCl, which suggests that the isolate is halotolerant. Similarly, *P. kudriavzevii* CMGB-M6 showed the maximum DO value in the presence of 0.5% NaCl, maintaining significant growth rates even at higher concentrations (up to 10%). *P. kudriavzevii* is known for its intrinsic ability to withstand particularly high salt concentrations (above 10% NaCl), thermal stress (temperatures above 45 °C), or extreme pH values (pH 1.0) due to a cross-protection mechanism [57]. A study by Matsushika et al. [58] revealed the existence of a gene, IoGAS1, similar to the GAS gene from *S. cerevisiae*, that encodes a key enzyme involved in cell wall assembly and whose overexpression facilitates the maintenance of the cell wall integrity in the presence of ionic stress induced by high salt concentrations or low pH values. A study performed by Mukherjee et al. [59], designed to select a non-*Saccharomyces* strain with better resistance to stress conditions associated with second-generation bioethanol fermentation showed that 3 out of four newly isolated *M. pulcherrima* strains can tolerate up to 8.9% NaCl (1500 mM). In the same study, it is presented that some *P. kudriavzevii* isolates (2 out of 7) or *T. delbrueckii* isolates (1 out of 12) can tolerate high concentrations of NaCl (up to 1500 mM). Regarding the ability of *T. delbrueckii* isolates to tolerate saline stress conditions, some papers suggest that this is more of a strain-dependent feature [42,60]. From this point of view, the *T. delbrueckii* CMGB-M5 shows good potential for industrial use.

An interesting fact was observed in the case of *H. uvarum* CMGB-M1. Although the isolate can tolerate up to 5% NaCl, at lower concentrations (2% NaCl), the growth rate decreased. This might be due to a dual mechanism of resistance to NaCl-induced stress. At concentrations lower than 2%, the response mechanism is a general one, while at higher concentrations, specific cellular response mechanisms are probably activated. Its ability to tolerate even 5% NaCl concentration is an important feature for members of this species, as previous studies have showed that *H. uvarum* has limited tolerance to saline stress [59,61]

The *S. cerevisiae* isolates MD3 and MS1-1 were stimulated by low concentrations of NaCl (0.5%) and tolerated up to 5% NaCl. However, it is noted that *S. cerevisiae* MS1-1 isolate can tolerate even 8% NaCl. Regarding the isolates from *S. cerevisiae* species, the study conducted by Subodinee et al. [62] confirms that the isolation of new strains from traditional beverages offers the possibility of obtaining highly stress-tolerant strains. In the above-mentioned study, researchers isolated new *S. cerevisiae* strains that tolerate up to 13% NaCl.

##### Influence of Different pH Values

In order to determine the impact of pH value variation on microbial growth, we cultivated each isolate in the presence of acidic and alkaline growth conditions. 

**Figure 7:**
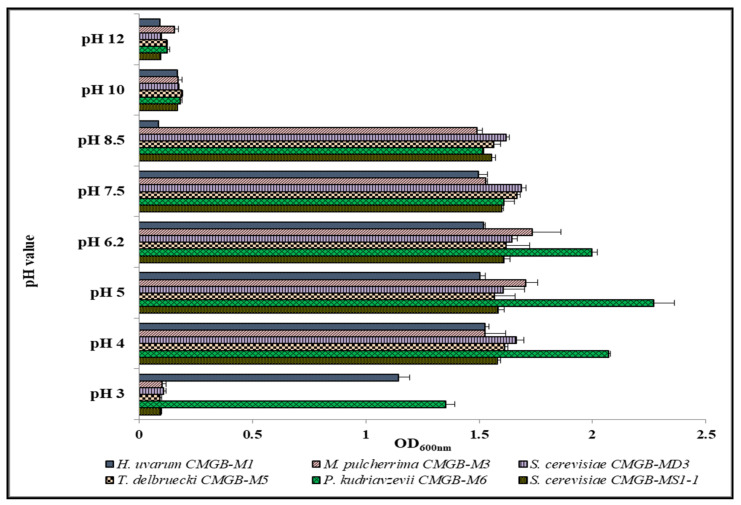
Growth rate of yeast strains isolated from wort sediment after 24 h of cultivation at different pH values.

The results showed that the optimal pH range tolerated by all our six isolates was 4.0–8.5 (Figure 7), except for *H. uvarum* CMGB-M1, for which no growth was observed at pH 8.5. At lower values (pH 3.0), significant growth was only recorded in the isolates *H. uvarum* CMGB-M1 and *P. kudriavzevii* CMGB-M6. The tolerance of *H. uvarum* CMGB-M1 to highly acidic environments it is important since another study reported that low pH values inhibit growth of the members of this species [61]. In the case of *P. kudriavzevii* CMGB-M6, it is not surprising that this strain showed a high ability to grow at low pH values since members of this species are well-known as being highly tolerant to stress conditions. Another study reported *P. kudriavzevii* strains isolated from grapes, peaches, and palm sludge as being able to grow even at pH 2.0 [54]. Both *S. cerevisiae* isolates (MS1-1 and MD3) showed a high ability to grow even at pH 8.5, which is surprising since members of this species are known for being inhibited by pH values higher than 8.0 [63].

Therefore, the determination of the resistance profile to different stress conditions associated with industrial processes revealed that the newly isolated yeasts present cellular response mechanisms that allow them to develop even in variable cultivation conditions, thus making them an invaluable microbial resource for different industrial processes.

#### 3.2.2. Confirmation of Safety Status; Synthesis of Virulence and Pathogenicity Factors

**Figure 8:**
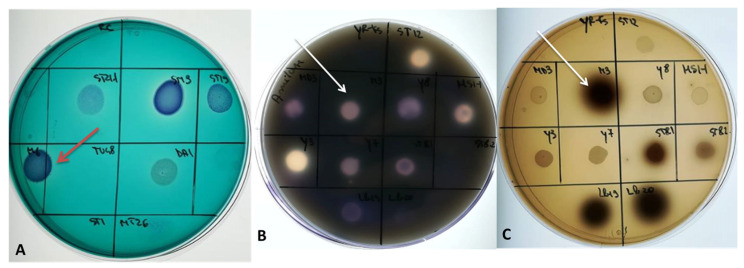
Phenotypic analysis of soluble virulence factors produced by M6 (red arrow) and M3 isolates (white arrow) ((**A**)—DNases; (**B**)—amylolytic enzymes; (**C**)—Siderophore-like compounds).

In the present study, a qualitative screening was performed regarding the secretion of the main hydrolytic enzymes affecting the tissues of a host cell. Thus, the yeast isolates were cultivated on specific media, and the secretion capacities of caseinases, amylases, gelatinases, hemolysins, phospholipases, and DNases were determined. In addition, the capacity of iron ions to accumulate from the environment by means of intermediate compounds such as siderophores was also monitored (Figure 8).

According to Table 4, none of the tested isolates produced gelatinases, phospholipases, or hemolysins. Regarding the caseinases’ secretion capacity, a rather weak positive result was recorded only in the case of *M. pulcherrima* CMGB-M3, but it was characterized by a rather weak secretion yield of these enzymes. The secretion of amylolytic enzymes was common among the analyzed yeast isolates. More precisely, four out of the six isolates produced amylases in the first 48 h after inoculation. 

The production of DNase was recorded only for *P. kudriavzevii* CMGB-M6. Some members of the *M. pulcherrima* species are described as being able to secrete DNA degrading enzymes [64] but in the case of *M. pulcherrima* CMGB-M3, the halo surrounding the colony appeared after more than 48 h so its degrading ability cannot be considered as an indicator of pathogenicity. Another frequently investigated virulence factor is the ability of yeasts to synthesize siderophores responsible for chelating the iron ions from the environment during different phases of tissue infection. Although it is frequently used to discriminate *Enterococcus* and *Streptococcus* bacterial strains, the bile aesculin agar medium can be successfully used to determine the ability of yeasts to hydrolyze aesculin and chelate the resulting iron ions via siderophores. According to Table 4, the isolates *H. uvarum* CMGB-M1 and *M. pulcherrima* CMGB-M3 produced siderophores, while for *T. delbrueckii* CMGB-M5, the positive response was recorded after several days of incubation. 

Another characteristic of strains with pathogenic potential is their ability to form biofilms. The first stage in the formation of the microbial biofilm is adhesion to the inert substrate. In the present study, a specific crystal violet staining was performed to quantify the number of cells that adhered to the substrate within 72 h. The results revealed that none of the analyzed yeasts adhered to the inert substrate, with the OD values recorded at 490 nm being less than 0.1.

The morpho-genetic dimorphism represented by the transition from vegetative propagation by budding to the pseudohyphal/hyphal form is another characteristic often encountered among yeasts with pathogenic potential. Although this behavior is most common among strains of the genus *Candida*, we analyzed all our yeasts regardless of the species to which they belong. Thus, after 3 h of cultivation on specific RPMI 1640 medium, no dimorhic changes of cell morphology were observed.

In conclusion, none of our isolates is characterized by all the virulence and pathogenicity factors analyzed and the positive results obtained might rather indicate their potential adaptation to the habitat of origin than a specific pathogenic character. In addition, except for *P. kudriavzevii,* none of the species to which our isolates belong, are known to be responsible for generalized infections in humans or animals. In the case of *P. kudriavzevii*, there are some studies regarding a possible pathogenicity associated with its teleomorphic form, *C. krusei* [65,66]. Furthermore, a study carried out by Zajc et al. [29] supports the idea that performing screening tests similar to those described in the present work, except for hemolysins, allows the identification of yeasts with potential application in biocontrol. The main idea is the existing overlap between the characteristics associated with pathogenicity and those associated with antimicrobial activity. In this context, amylases or caseinases producing isolates can be used on a large scale in the food and chemical-pharmaceutical industries, while the yeasts producing siderophore-like compounds can prove useful in studies on the control of pathogenic eukaryotic and prokaryotic microorganisms.

### 3.3. Mechanisms of Antimicrobial Activity against Candida Strains

Yeast survival in different ecological niches requires both the development of specific stress resistance mechanisms and the removal of competing microorganisms [67]. The antimicrobial activity of yeasts has important applications in biomedicine as anti-pathogenic agents [68,69]. These applications derive from their potential to inhibit the growth of microbial strains responsible for localized or systemic infections in humans and animals. Yeast strains from the genus *Candida* are most commonly associated with human infections, among which the species *C. albicans*, *C. krusei*, *C. glabrata*, *C. parapsilosis,* and *C. tropicalis* stand out. They are opportunistic pathogens and can cause both superficial and systemic infections [70]. Members of these species are particularly resilient and can adapt to particularly harsh environmental conditions. Considering the increase in the number of infections caused by *Candida* strains, the development of an alternative strategy to combat them is desirable [71]. There are two possible approaches for the development of products with antimicrobial action based on yeasts: using non-pathogenic cultures with proven antimicrobial characteristics or various metabolites with potential antimicrobial activity. 

The antagonistic action of the yeasts is most commonly based, in general, on competition for nutrients from the environment and on the synthesis of secondary metabolites such as toxins, enzymes, and volatile compounds that act on the potentially sensitive strain [72]. 

The studies concerning the antimicrobial potential of our yeasts aimed to determine the mechanism of activity against *Candida* species frequently associated with both food contamination and human infections. 

#### 3.3.1. Competition for Nutritive Substrates

The antimicrobial activity of yeasts based on substrate competition reflects mainly the relationships between microorganisms from the same ecological niche. Starting from this idea, we can extrapolate their importance in industrial processes and biomedicine as an indicator of the ability of the yeasts to limit the expansion of competing strains. The tests were performed using YMA medium, originally described for the determination of the antimicrobial activity of *M. pulcherrima* strains based on the competition for iron ions in the culture medium. Iron is a trace element involved in almost all cellular processes carried out in eukaryotic cells, acting as a redox cofactor [73]. 

From the six yeasts, only *M. pulcherrima* CMGB-M3 tested positive for competition for iron ions against *C. parapsilosis* CBS 604, *C. krusei* CMGB94, and *C. albicans* ATCC10231 (Table 5). This is not surprising, since the main mechanism of antimicrobial activity for the species belong to the genus *Metschnikowia* consists in secretion of pulcherrimic acid which chelates the iron ions fromthe environment forming a reddish-brown pigment called pulcherrimine. Moreover, although the mechanism is well described for *M. pulcherrima*, it seems to be present in other species such as *M. sinensis, M. shaxiensis,* and *M. fructicola,* which also approach such a strategy [74].

#### 3.3.2. Synthesis of Killer Toxins

Being involved in numerous fermentation processes, yeasts develop optimally when grown in the presence of a slightly acidic environment. Most yeast species with antimicrobial activity, including *S. cerevisiae*, have the ability to synthesize compounds with an antagonistic role when grown in the presence of an acidic environment. The K (killer) medium used in the present study was originally described for screening the killer activity in *S. cerevisiae*. The K medium contains methylene blue, which allows visualization of halos and inhibition zones occurring at the level of the sensitive cells on the substrate. 

According to Table 6, the antimicrobial activity of the six yeasts was influenced both by the incubation time and the potentially sensitive *Candida* strain used as a substrate. Best results were obtained for *M. pulcherrima* CMGB-M3, *T. delbrueckii* CMGB-M5, and *S. cerevisiae* CMGB-MD3. Thus, *M. pulcherrima* CMGB-M3 inhibited the growth of *C. krusei*, *C. albicans,* and *C. parapsilosis* strains. This species is well known for its ability to inhibit the growth of other yeasts through substrate competition mediated by pulcherriminic acid, whose production is strongly promoted by low pH [75,76]. Even so, it seems that the antagonistic activity of a *M. pulcherrima* strain is not based exclusively on substrate competition [32], and, in certain situations, some strains maintain their inhibitory activity even after pulcherriminic acid production is stopped [77]. Hence, their inhibitory action might also be due to other compounds secreted in the culture medium. Thus, several studies shown that different *M. pulcherrima* strains secrete extracellular lytic enzymes (chitinase and glucanase) [78] or volatile organic compounds such as ethyl acetate [79] with strong inhibitory activity over filamentous fungal strains or pathogenic *Candida* [80]. Moreover, some studies describe the possible production of killer toxins by different *M. pulcherrima* strains. Thus, *M. pulcherrima* strains were reported as exerting killer activity against *P. guilliermondii* and *P. membraniefaciens* spoilage yeasts [81] or *M. luteus* [82]. It seems that the killer activity is promoted in the presence of 1% NaCl [81] or when the producing strain is cultivated in the presence of glycerol as a carbon source at 20 °C and a neutral pH value [82].

Although the killer character of *P. kudriavzevii* strains has only recently been identified and the studies are at an early stage, they represent an important topic since the secreted toxin can inhibit the growth of bacterial pathogens such as *S. aureus, E. coli, Klebsiella sp*., *P. aeruginosa*, and *P. alcaligenes*. The killer toxin shows maximum activity at 30 °C and is stable both at low temperatures and at temperatures exceeding 30 °C (for a maximum of 3 days).Tthe presence of Mn^2+^, Fe^2+^, or Zn^2+^ ions causes a reduction in the activity of the killer toxin, while no effect was observed in the case of Mg^2+^ or Ca^2+^ ions. Regarding the toxin’s resistance to pH variation, the toxin’s stability range is between 4.6 and 7.0, with maximum yield at pH 5.0. Although the toxin proved particularly effective against the bacterial species mentioned above, no antifungal activity was determined [83]. However, the isolate *P. kudriavzevii CMGB-M6* showed limited activity only against *C. parapsilosis* CBS604 after the first 3 days of incubation. 

Good antimicrobial activity was observed for *S. cerevisiae* CMGB-MD3. This isolate inhibited growth of pathogenic and reference *C. krusei* strains, presenting limited activity against *C. parapsilosis* CBS604, pathogenic *C. albicans* ATCC10231, and *C. albicans* CMGB Y12 after 3 days of incubation. The isolate *S. cerevisiae* CMGB-MS1-1 did not present antimicrobial activity. This is not surprising, since the killer toxins are synthesized only by some strains as a competition mechanism for survival in various ecological niches. Moreover, the *S. cerevisiae* killer character is highly heterogenous in terms of encoding genetic material, chemical structure, and mode of action [84].

There are four types of *S. cerevisiae* killer toxins, namely K1, K2, K28, and Klus. The mature K1, K2, and K28 toxins are α/β heterodimers stabilized by disulfide bridges that recognize specific binding sites present in the cell wall of susceptible yeast cells. K1 and K2 toxins recognize and bind without ATP consumption to the β-1,6 D-glucan receptor, while the K28 toxin specifically recognizes α-1,3-mannanoproteins. Both K1 toxin and K2 toxin cause the death of the sensitive cell by increasing the membrane permeability for H+ ions and the uncontrolled release of K+ ions but also of other molecules such as amino acids or glucose [85]. The K28 toxin, after binding to the specific receptor, is internalized by endocytosis and reaches the nucleus of the susceptible cell, where it disrupts the division process, causing cell arrest in the G1 phase and subsequent death of the susceptible yeast cell [86]. Klus toxin was recently identified among *S. cerevisiae* strains from spontaneously fermented wine products from Spain. Although its mechanism of action is not fully elucidated, Klus toxin shows a lower degree of specificity, exhibiting wide intra and interspecific antimicrobial activity. Thus, the Klus protein shows high potential for use in alcoholic fermentation for limiting contamination with microorganisms that cause a decrease in the quality of fermented alcoholic beverages [87,88]. 

The isolate *T. delbrueckii* CMGB-M5 was able to inhibit the growth of both *C. krusei* strains after 3 days of incubation, making it more efficient against the pathogenic strain *C. krusei* CMGB-Y8. Members of the *T. delbrueckii* species are frequently encountered in the late stages of wine fermentation and are known for their high ethanol tolerance. Some of them are able to secrete killer toxins with high potential for wine spoilage biocontrol [89]. Thus, the TdKT toxin is highly tolerant to pH and temperature variations and maintains its activity at different ethanol, glucose, and sulphur dioxide concentrations [90]. The toxin usually binds to cell wall receptors such as β-1,6-glucans, mannans, or chitin of the sensitive cells, followed by strong glucanase and chitinase activity. The overall effect is disruption of the cell wall structure, which leads to apoptosis and necrosis [91].

It is interesting to notice that the two *C. krusei* strains were the most sensitive to the killer activity of *M. pulcherrima CMGB-M3, T. delbrueckii CMGB-M5,* and *S. cerevisiae CMGB-MD3*. The explanation resides, most probably, in the specific mechanism of action of the killer toxin produced by the three yeast strains correlated with the structure of the *C. krusei* cell wall bearing the receptor for the killer toxin. 

Further investigations focusing on a broader range of pathogenic microorganisms and different cultivation parameters are expected to offer a more comprehensive understanding of the antimicrobial activity of the six yeasts.

## 4. Conclusions

The microbiota involved in the spontaneous fermentation of drinks and food represent an invaluable resource for the optimization of industrial processes. In the present study, six isolates of yeasts originating from wort sediment were isolated and taxonomically identified. Their characterization emphasized their increased resistance to stress factors associated with industrial processes and their non-pathogenic character. Moreover, the isolates have the advantage of being isolated from local products, an absolutely necessary basis for the conservation of microbial biodiversity. These features recommend them as valuable resources for the possible development of starter cultures for artisanal fermentation processes. By means of this study, the first steps in the characterization of the isolates as potential starter cultures were achieved, for which a much more detailed characterization is required. Finally, the antimicrobial activity could be used in the food industry for the control of microbial contamination of fermentation processes and in biomedicine against infections with *Candida* strains as an etiological agent.

## Figures and Tables

**Table 1:** Conventional test results obtained for yeasts isolated from wine wort and automatized taxonomical identification using the Biolog Microbial ID System and Bruker Maldi-TOF.

Conventional Tests		MD3	M1	M3	M5	MS1-1
Ascopores	Non-fermentative carbon sources (10% CaCO_3_ Agar	+	-	-	-	+
	5% Malt agar	NT	+	-	+	NT
Urease	Christensen’s Urea Agar +0.1% glucose	-	-	+	-	-
	Difco Urea Broth	NT	NT	-	NT	NT
Cycloheximide	0.01%	-	W	-	-	-
	0.1%	-	-	-	-	-
Temperature	20 °C	+	+	+	+	+
	28 °C	+	+	+	+	+
	37 °C	+	W	+	+	+
	42 °C	+	-	-	-	+
Osmotic stress	Glucose 50%	+	+	+	+	+
	Glucose 60%	+	+	+	+	+
Carbon aerobic assimilation tests	D-Glucose	+	+	+	+	+
	D-galactose	W	-	+	-	-
	Xylitol	-	-	+	-	-
	Raffinose	-	-	-	-	W
Nitrogen assimilation tests	L-lysine	-	+	+	+	-
	Potassium nitrate	-	-	-	-	-
	Sodium nitrite	-	-	-	-	-
	Ammonium sulphate	+	+	+	+	+
Biolog Microbial ID System (Figure S1)		*S. boulardii*	No id	*M. pulcherrima*	No id	*S. cerevisiae*
Bruker Maldi-TOF MS		*S. cerevisiae*	*H. uvarum*	*M. pulcherrima*	No id	*S. cerevisiae*

- Negative result; + Positive result; D delayed (positive result recorded after at least 1 week from inoculation); W weak; NT—not tested.

**Table 2:** Conventional taxonomy test results for reference strains [18,20].

Conventional Tests		*S. cerevisiae* CBS380	*H. uvarum* CBS279	*M. pulcherrima* CBS5534	*T. delbrueckii* CBS133
Ascospores	Non-fermentative carbon sources (10% CaCO_3_ Agar/Acetate agar)	+	NT	NT	NT
	5% Malt Agar		+	-	+
Urease		-	-	-	-
Cycloheximide	0.01%	-	+	-	-
	0.1%	-	+/-	-	-
Temperature	20 °C	+	+	+	+
	28 °C	+	+	+	+
	37 °C	+/-	-	-	+/-
	42 °C	+/-	-	-	-
Osmotic stress	Glucose 50%	+/-	+/-	+/-	+
	Glucose 60%	-	-	+/-	+/-
Carbon aerobic assimilation tests	D-Glucose	+	+	+	+
	D-galactose	+/-	-	+	+/-
	Xylitol	-	-	+	+/-
	Raffinose	+/-	-	-	+/-
Nitrogen assimilation tests	L-lysine	-	+	+	+
	Potassium nitrate	-	-	-	-
	Sodium nitrite	-	-	-	+/-
	Ammonium sulphate	+	+	+	+

- Negative result; + Positive result; D: delayed (positive result recorded after at least 1 week from inoculation); W: weak; NT: not indicated in the monographs.

**Table 3:** Size of amplicons and restriction fragments obtained for the ITS1-5.8S-ITS2 region for yeasts isolated from wine wort and species of interest described in the literature (nd: not determined).

Isolate	Amplicon (bp)	*Cfo*I (bp)	*Hae*III (bp)	*Hinf*I (bp)	*Msp*I (bp)	Reference
MD3	850	135; 350; 350	135; 175; 225; 325	135; 350; 350	130; 700	
MS1-1	850	140; 350; 350	135; 175; 20; 320	135; 350; 350	130; 700	
*S. cerevisiae*	850	nd	nd	nd	130; 740	[43]
*S. cerevisiae*	860	nd	nd	125; 370	nd	[44]
*S. cerevisiae*	880	nd	150; 180; 230; 320	155; 365	nd	[45]
*S. cerevisiae*	880	nd	150; 180; 240; 310	40; 120; 350	120; 750	[46]
*S. cerevisiae*	880	365; 385	130; 180; 230; 320	180; 350; 360	nd	[47]
*S. cerevisiae*	850	150; 325; 375	125; 230; 495	110; 365; 375	nd	[48]
M5	775	100; 150; 225; 350	750	375; 400	775	
*T. delbrueckii*	798	102; 139; 220; 327	800	160; 190; 360	nd	[49]
*T. delbrueckii*	800	100; 150; 220; 330	798	380; 410	nd	[49]
M3	390	100; 200	100; 290	190; 200	50; 120; 210	
*M. pulcherrima*	390	100; 210	100; 285	190; 200	nd	[50]
M1	725	100; 315; 320	715	65; 135; 160; 325	715	
*H. uvarum* CBS 314	745	105; 315; 324	747	62; 155; 188; 342	nd	[49]
*H. uvarum* CECT 1444	760	105; 315; 320	760	180; 200; 360	nd	[49]
*H. uvarum* NCYC2739	750	105; 310; 320	750	180; 200; 350	nd	[49]
*H. uvarum* CBS 104	745	105; 315; 324	745	70; 150; 180; 330	nd	[49]

**Table 4:** Evaluation of the ability to produce soluble virulence factors.

Isolates	Caseinase	Amilase	Gelatinase	Hemolysine	Phospholipase	Siderophore-*like* Compounds	DN-Ase
*H. uvarum CMGB-M1*	-	-	-	-	-	+	-
*M. pulcherrima CMGB-M3*	W	+	-	-	-	+	D
*T. delbrueckii CMGB-M5*	-	+	-	-	-	D	-
*P. kudriavzevii CMGB-M6*	-	+	-	-	-	-	+
*S. cerevisiae CMGB-MD3*	-	-	-	-	-	-	-
*S. cerevisiae CMGB-MS1-1*	-	+	-	-	-	-	-

Legend: “-” negative result; “+” positive result, “D”—the positive result appeared after the moment of determination; “W”—the positive result is recorded on the day intended for monitoring, but the intensity of the reaction is lower.

**Table 5:** Demonstration of antimicrobial activity based on substrate competition against *Candida* strains at 3 and 7 days of incubation on YMA medium at 28 °C.

Isolates		*C. parapsilosis* CBS 604	*C. parapsilosis* CMGBY3	*C. krusei* CMGB 94	*C. krusei* CMGB-Y8	*C. albicans* ATCC10231
*H. uvarum CMGB-M1*	3 days	-	-	-	-	-
	7 days	-	-	-	-	-
*M. pulcherrima CMGB-M3*	3 days	+	-	+	-	-
	7 days	+	-	+	-	++
*T. delbrueckii CMGB-M5*	3 days	-	-	-	-	-
	7 days	-	-	-	-	-
*P. kudriavzevii CMGB-M6*	3 days	-	-	-	-	-
	7 days	-	-	-	-	-
*S. cerevisiae CMGB-MD3*	3 days	-	-	-	-	-
	7 days	-	-	-	-	-
*S. cerevisiae CMGB-MS1-1*	3 days	-	-	-	-	-
	7 days	-	-	-	-	-

Legend: “+” 2–3 mm halo with medium luminous intensity; “++” halo of 2–3 mm with strong luminous intensity; “-” lack of halo.

**Table 6:** Highlighting the antimicrobial activity in the presence of low pH values against *Candida* strains at 3 and 7 days of incubation on medium K.

Isolates	*C. parapsilosis* CBS 604		*C. parapsilosis* CMGBY3		*C. krusei* CMGB94		*C. krusei* CMGB Y8		*C. albicans* ATCC 10231	
	3 Days	7 Days	3 Days	7 Days	3 Days	7 Days	3 Days	7 Days	3 Days	7 Days
*H. uvarum CMGB-M1*	-	-	-	-	-	-	-	-	-	-
*M. pulcherrima CMGB-M3*	-	-	-	+	+	++	++	++	+	+
*T. delbrueckii CMGB-M5*	-	-	-	-	+	-	++	+	-	-
*S. cerevisiae CMGB-MD3*	+	-	-	-	+	+++	++	++	+	-
*S. cerevisiae CMGB-MS1-1*	-	-	-	-	-	-	-	-	-	-
*P. kudriavzevii CMGB-M6*	+	-	-	-	-	-	-	-	-	-

Legend: “+” 2–3 mm halo with medium luminous intensity; “++” halo of 2–3 mm with strong luminous intensity; “+++” 5–6 mm halo; “-” lack of halo.

## Data Availability

Yeast isolates are available from the authors.

## Supplementary Materials

The following supporting information can be downloaded at: https://www.mdpi.com/article/10.3390/microorganisms11010037/s1, Figure S1: Appearance of the phenotypic phylogeny dendrograms generated using the Biolog Microbial ID System (MicroStation™ System MicroLog version 4.2) for the strains: A-MS1-1; B-M3; C-MDR; whose percentage of similarity identified was >95%; Figure S2: Urease test results using Christensen’s Urea Agar supplemented with 0.1% glucose after 24 h of incubation at 28°C (A-*Y. lipolytica* CMGB 32; B-*R. mucilaginosa* CMGB-G1; C-*S. cerevisiae* CMGB-RC; D-M3); Figure S3: Urease test results using Difco Urea Broth 24 h of incubation at 28 °C (A-*R. mucilaginosa* CMGB-G1- positive control; B-*S. cerevisiae* CMGB-RC-negative control; C-M3; D-*Y. lipolytica* CMGB 32; 1-the initial aspect of the test tube after inoculation; 2-the aspect of the test tube after 14 h of incubation at 37 °C); Table S1: Metabolic profile obtained using Biolog Microbial ID System after 72 h of incubation at 28 °C.
