# Supplementary material for: Molecular and Physiological Diversity of Indigenous Yeasts Isolated from Spontaneously Fermented Wine Wort from Ilfov County, Romania
Source: Microorganisms. 2022 Dec 22;11(1):37. doi: 10.3390/microorganisms11010037 (PMC9861077; doi:10.3390/microorganisms11010037)
Supplement: Supplementary file 1 [file microorganisms-11-00037-s001.zip › microorganisms-2057157-supplementary.pdf]

Supplementary file

Molecular and physiologic diversity of indigenous yeasts isolated from spontaneusly fermented wine wort from Ilfov county, Romania

Viorica Maria Corbu [1,*], Ortansa Csutak [1]

[1] Department of Genetics, Faculty of Biology, University of Bucharest, 060101, Bucharest, Romania;

* Correspondence: viorica.corbu@yahoo.com;

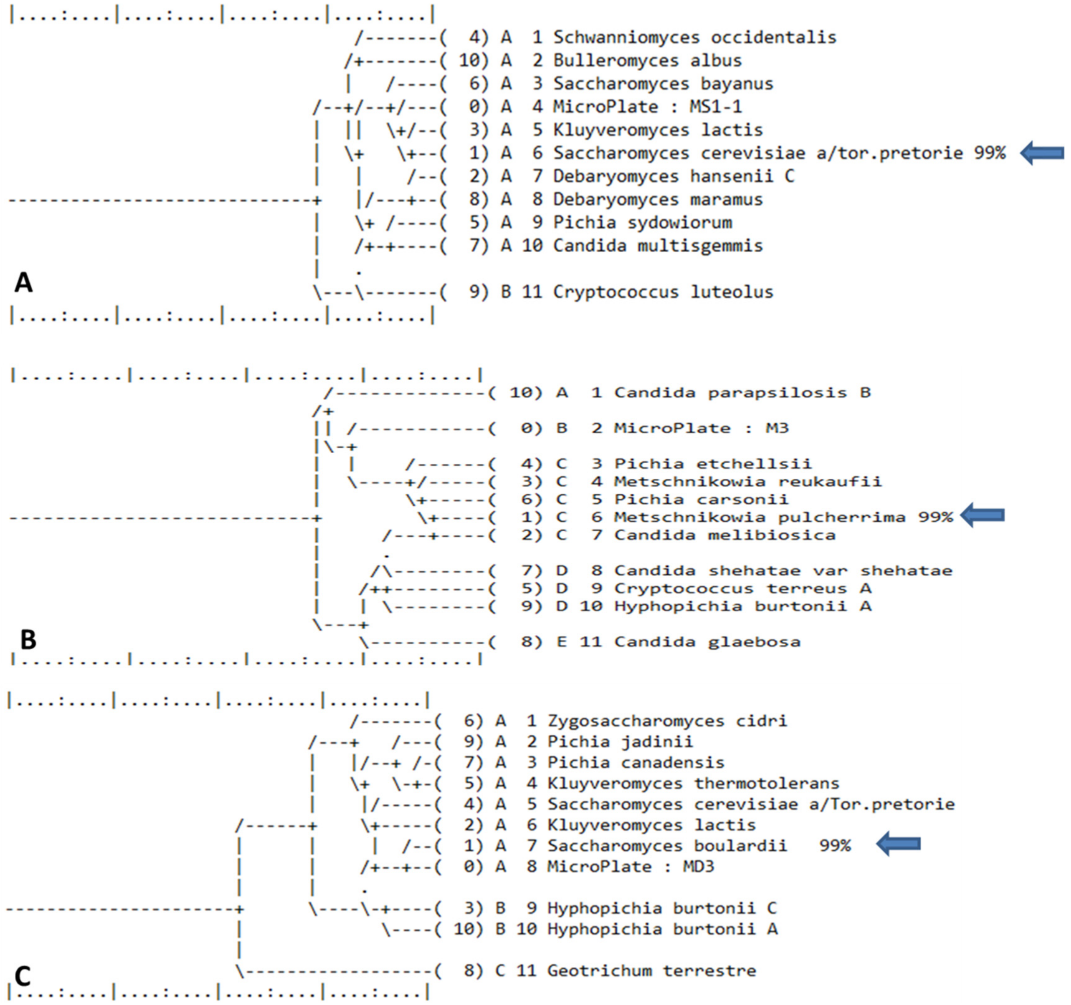

Figure S1. Appearance of the phenotypic phylogeny dendrograms generated using the Biolog Microbial ID System (MicroStation™ System MicroLog version 4.2) for the strains: A- MS1-1; B-M3; C-MDR; whose percentage of similarity identified was >95%

Table S1. Metabolic profile obtained using Biolog Microbial ID System after 72 hours of incubation at 28°C

		M1	M3	MD3	MS1-1	M5
	**Oxidation tests results**					
1	Water	M	M	M	M	M
2	Acetic acid	S	-	-	-	-
3	Formic acid	S	-	S	S	S
4	Propionic acid	+	-	S	-	S
5	Succinic acid	S	B	-	-	S
6	Succinic acid mono-methyl ester	S	B	-	-	-
7	L-Aspartic acid	+	-	-	-	-
8	L-Glutamic Acid	S	B	-	-	-
9	L-proline	S	+	-	-	-
10	D-Gluconic Acid	B	+	-	-	B
11	Dextrin	S	-	-	-	-
12	Inulin	+	+	+	S	+
13	D-cellobiose	+	+	S	S	-
14	Gentiobiose	+	+	S	S	S
15	Maltose	S	B	+	+	S
16	Maltotriose	S	+	S	S	-
17	D-melezitose	S	+	S	S	S
18	D- melibiose	S	S	S	S	S
19	Palatinose	S	+	S	S	S
20	D-raffinose	S	S	B	-	B
21	Stachyose	S	S	-	-	B
22	Sucrose	S	+	+	B	+
23	D-trehalose	S	B	B	B	B
24	Turanose	S	B	B	B	-
25	N-acetyl D-glucosamine	S	+	S	S	S
26	α-D glucose	+	+	+	+	+
27	D-galactose	B	B	+	+	S
28	D-psicose	-	B	S	S	-
29	L-sorbose	S	+	S	S	-
30	Salicin	+	B	S	S	S
31	D-mannitol	S	+	S	S	B
32	D-sorbitol	S	+	S	S	B
33	D-arabitol	S	-	S	S	-
34	Xylitol	S	+	S	S	B
35	Glycerol	S	-	S	S	-
36	Tween 80	S	S	S	S	-
	**Assimilation tests**					
37	Water	M	M	M	M	M

38	Fumaric acid	-	B	S	S	S
39	L-malic acid	S	+	S	S	-
40	Succinic Acid Mono-Methyl Ester	S	B	-	-	-
41	Bromosuccinic acid	-	B	S	S	S
42	L-glutamic acid	-	B	S	S	-
43	γ-aminobutyric acid	-	B	S	S	-
44	α-Ketoglutaric Acid	S	B	S	S	S
45	2-Keto-D-Gluconic Acid	+	+	S	S	-
46	D-gluconic Acid	+	+	S	S	-
47	Dextrin	-	-	S	S	-
48	Inulin	+	+	+	+	+
49	D-cellobiose	+	+	-	-	-
50	Gentiobiose	+	B	-	-	S
51	Maltose	S	+	+	B	S
52	Maltotriose	-	B	+	+	-
53	D-melezitose	-	+	-	-	S
54	D- melibiose	S	-	S	S	S
55	Palatinose	-	B	S	S	S
56	D-raffinose	S	-	+	-	B
57	Stachyose	S	S	B	-	B
58	Sucrose	-	B	+	+	+
59	D-trehalose	B	B	+	+	B
60	Turanose	S	B	+	+	-
61	N-acetyl D-glucosamine	S	B	S	S	S
62	D-glucosamine	S	B	S	S	-
63	α-D glucose	+	+	+	+	+
64	D-galactose	B	B	+	+	S
65	D-psicose	B	B	-	-	S
66	L-rhamnose	-	S	S	S	S
67	L-sorbose	S	B	S	S	S
68	α-methyl-D-glucoside	S	B	S	B	S
69	β-methyl-D-glucoside	+	B	S	S	S
70	Amygdalin	+	B	B	B	B
71	Arbutin	B	B	S	S	S
72	Salicin	B	B	-	-	-
73	Maltitol	B	B	S	S	S
74	D-Mannitol	S	+	S	S	B
75	D-sorbitol	S	B	S	S	B
76	Adonitol	-	B	S	S	S
77	D-arabitol	S	B	-	-	-
78	Xylitol	S	+	-	-	B
79	*i*-erythritol	S	S	-	-	S
80	glycerol	S	B	-	-	-

81	Tween 80	S	S	S	S	S
82	L-arabinose	B	S	-	-	S
83	D-arabinose	B	S	-	-	S
84	D-ribose	+	-	-	-	-
85	D-xylose	+	+	-	-	-
86	Succinic acid Mono-methyl ester plus D-xylose	+	+	B	S	B
87	N-acetyl-L-glutamic acid plus D-xylose	+	B	B	S	B
88	Quinic acid plus D-xylose	+	+	-	-	S
89	D-glucuronic acid plus D-xylose	+	B	S	S	S
90	Dextrin plus D-xylose	-	B	S	S	S
91	α-D-Lactose plus D-Xylose	-	B	S	S	S
92	D-melibiose plus D-xylose	-	B	S	S	S
93	D-galactose plus D-xylose	+	+	+	B	S
94	m-inositol plus D-xylose	-	B	-	-	S
95	1,2 propanediol plus D-xylose	-	B	B	-	-
96	Acetoin plus D-xylose	B	B	-	-	S

+ positive; - negative; B- borderline; S- no positive or negative result (the value obtained was less than the one recorded for control well); M-control well

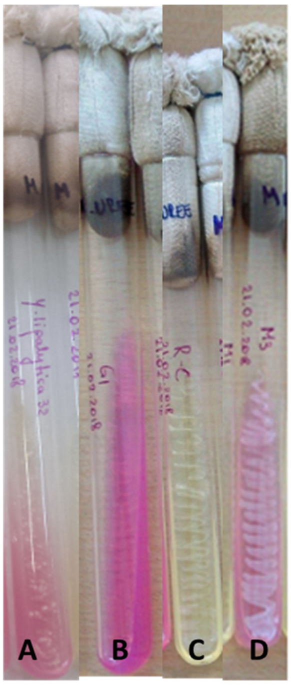

Figure S2. Urease test results using Christensen's Urea Agar supplemented with 0,1% glucose after 24 hours of incubation at 28°C (A-*Y. lipolytica* CMGB 32; B- *R. mucilaginosa* CMGB-G1; C- *S. cerevisiae* CMGB-RC; D- M3)

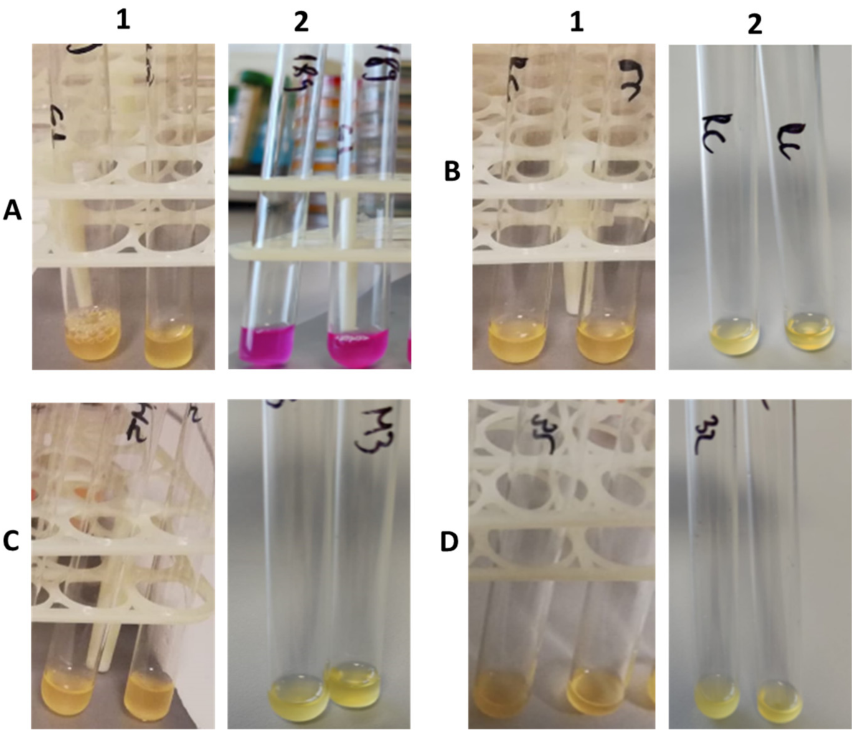

Figure S3. Urease test results using Difco Urea Broth 24 hours of incubation at 28°C (A- *R. mucilaginosa* CMGB-G1-positive control; B- *S. cerevisiae* CMGB-RC-negative control; C- M3; D-*Y. lipolytica* CMGB 32; 1- the initial aspect of the test tube after inoculation; 2-the aspect of the test tube after 14 hours of incubation at 37°C)
